# Enhance PARPi Application beyond BRCA-Mutant Breast Cancer (BC): Facts Are Facts

**DOI:** 10.3390/jcm9082377

**Published:** 2020-07-25

**Authors:** Tania Di Raimo, Francesco Angelini

**Affiliations:** Clinical and Experimental Pharmacology, Centro di Riferimento Oncologico di Aviano (CRO) IRCCS, 33081 Aviano (PN), Italy; tania.diraimo@gmail.com

Breast cancer (BC) represents one of the three most common neoplasia and the principal worldwide leading cause of death among women [1,2,3]. Generally, the term BC refers to a heterogeneous disease with multiple intrinsic tumour subtypes with distinctive histopathological and biological characteristics that reflects different clinical outcomes and therapeutic strategies [4,5]. Furthermore, the possibility to develop one of them is directly related to many factors, such as genetics, aging and life habits [6,7]. Nowadays, it has been amply demonstrated how the expression of specific markers, such as estrogen and progesterone receptors (ER and PR), human epidermal growth factor receptor 2 (HER-2) and the proliferation index (Ki-67), is correlated with both BC intrinsic subtype classification and the relative prognosis [8]. The canonical molecular classification divided BC into two principal subfamilies, ER-positive and ER-negative [9]. In the first subfamily, the LUMINAL A (ER^+^PR^+^HER2^−^Ki67^−^) and LUMINAL B (ER^+^PR^+/−^Her2^+/−^Ki67^+^) subgroups that represent the most common subtypes among BC are included. The ER-negative subfamily includes two principal subgroups, the HER2 OVER-EXPRESSED (ER^−^PR^−^Her2^+^Ki67^+^) and the so-called BASAL LIKE, that represents 15% of BC and is characterized by expression patterns including a lack or low expression of ER, PR and HER2 in addition to a high expression of basal markers and Ki67. TRIPLE NEGATIVE BC (TNBC), characterized by the absence of the principal three biological marker expressions, represents 60–90% of basal-like BC cases [10]. TNBC is a very heterogeneous subgroup which comprises a further six subclasses characterized by a high proliferation rate, an increase in basal/myoepithelial cells-related cytokeratins (CKs) and epidermal growth factor receptor (EGFR) expression [11]. Furthermore, even if its heterogeneity is correlated with different prognosis and severity levels, the high percentage of TNBC patients presents a worse clinical outcome, a shorter relapse-free period and a strong possibility to develop metastasis [12].

In normal cells, single-strand break (SSB) or double-strand break (DSB) repair pathways are responsible for the detection and repair of DNA damages, the so-called DNA Damage Response (DDR) [13]. In comparison to SSB, DSB is more cytotoxic for cells, because of the loss of large chromosomal regions, and their repair systems include the homologous recombination (HR) and nonhomologous end joining (NHEJ) mechanisms. It has been widely demonstrated that breast cancer susceptibility gene 1 (BRCA1) and breast cancer susceptibility gene 2 (BRCA2) play a role in the HR repair (HRR) system for the DSB in DNA and ensure genomic stability, making them tumour suppressor genes [14,15]. When mutations in BRCA1 and/or BRCA2 occur, cells are more inclined to develop genetic alterations, due to an impair in HRR pathway, leading to irregularities in the DNA synthesis and, thus, increasing the risk of developing cancer [16]. As a matter of fact, it has been observed that germline heterozygous mutations in BRCA1/2 genes increase the risk associated with developing breast cancer by 50–85% [17,18]. In addition, a great percentage of tumours arising in BRCA1 mutation carriers is more likely to be TNBC and to have a more aggressive phenotype and high-grade tumour, while BRCA2 tumours are often ER+ with a lower grade and high mitotic rates [15,19]. In this optic, BRCA1 and BRCA2 mutation status represent a significant prognostic value especially in those patients with a high risk of developing BRCA germline mutations [17,18,20]. Interestingly, it has been demonstrated that sporadic BCs can also present dysfunction in the HRR pathway, a phenotype called ‘’BRCAness”, due to mutations in BRCA1, BRCA2 and other HRR-associated genes, such as PALB2, BARD1, and RAD51D [21,22].

From a more mechanistic point of view, in the presence of an impaired HRR pathway, NHEJ is activated to repair DSB damages [23]. Differently from HRR, NHEJ is a rapid, low fidelity and error-prone mechanism able to create unsustainable chromosomal rearrangements detrimental for cell survival [24]. Therefore, SSB repair mechanisms could represent the most suitable alternative for HRR deficiency cells survival [25]. One of the principal effectors of SSB repair mechanisms is the Poly(ADP-ribose) polymerase (PARP) enzyme family able to covalently adding poly (ADP-ribose) chains onto target molecules, the so called PARylation [26]. PARP1 is one of the most abundant and well characterized members of the PARP family. It is mostly correlated with DNA damage repair, generating about the 90% of poly (ADP-ribose) chains after a DNA damage event, and it is also able to contribute to the HR pathways of DSB repair by promoting the recruitment of several effectors, including BRCA1 and BRCA2, to the DNA damage sites [13,26,27]. Taking into account what has been said so far, the discovery of small-molecule inhibitors of PARP (PARPi) that selectively target germline *BRCA* mutant (gBRCAm) cancer cells, leading to new therapeutic approaches to treat patients with BC is not surprising [28].

In their 2019 study entitled “PARP Inhibitors as a Therapeutic Agent for Homologous Recombination Deficiency in Breast Cancers”, Keung, Wu and Vadgama provide a deep analysis concerning the therapeutic potential of PARPi in BC cancer in addition to many other aspects strictly correlated with this issue [29]. As stated by authors, the principal aim of their work is to provide a basis for expanding the use of PARPi beyond BRCA-mutant BC, such as BRCAness and tumours harbouring loss-of-function mutations in HRR genes other than BRCA1/2. Starting from the only two currently FDA approved PARP inhibitors for gBRCAm metastic BC, *Olaparib* and *Talazoparib*, Keung et al., through a detailed review of scientific literature and ongoing clinical trials, make a point on the actual clinical development of PARPi as a single agent or in combination with chemotherapy, antiangiogenic agents, ionizing radiation or immunotherapy [29,30,31]. In the same way, authors analyze the current knowledge of the other three under evaluation PARPi: *Niraparib*, *Rucaparib* (both approved, together with *Olaparib*, by FDA and EMA for the treatment of ovarian cancers) and *Veliparib* [29,32]. Among the more than 150 completed, recruiting or current registered clinical trials involving the previously mentioned PARP inhibitors in BRCA mutated tumours, authors focus their attention on several studies concerning ovarian cancer that highlight the utility of PARPi also with non-BRCA-mutated tumours [29,33]. For example, in the randomized, double blind, placebo-controlled trial, phase-III trial NOVA (NCT01847274), it has been shown that patients receiving *Niraparib* present a longer median progression free survival (PFS) than those receiving the placebo, regardless of the presence or absence of gBRCAm or HR deficiency status [34]. Similar conclusions were obtained from the phase-II, open-label, multi-center study ARIEL2 (NCT01891344) in which HR deficiency patients with gBRCAm or wild type BRCA, with a high loss of heterozygosity (LOH), were able to respond to *Rucaparib* treatment [35,36]. This encouraging result paved the way to the single arm, open-label, multi-center phase-II study RUBY (NCT02505048) that is currently evaluating the efficacy of *Rucaparib* in patients with HER-negative metastatic BC with a BRCAness phenotype excluding gBRCA1/2m [37].

Generally, even if PARPi, now, have a clinical use restricted to a small category of patients with gBRCAm, there is no doubt that PARP inhibition represents a wider promising therapeutic strategy to treat different kinds of BC and a large amount of progress is being made to fully understand their potential. In this regard and as a result of their previous mentioned paper, Keung et al. recently publish an interesting work entitled “Response of Breast Cancer Cells to PARP Inhibitors Is Independent of BRCA Status” [29,38]. Here, authors examined the in vitro inhibitory effect of 13 different PARPi, including *Niraparib, Olaparib, Rucaparib, Talazoparib*, in 12 BC cell lines with and without BRCA-mutations using cell viability assays [38]. Once again, they demonstrated, but this time from a practical point of view, that the inhibitory activity of PARPi affects cell growth, independently from the BRCA status, suggesting the possibility that cell lines sensitive to PARPi might present defects on other HRR factors [38].

In conclusion, provided that further studies are required and essential to understand the overall biological meanings of PARP and its inhibition and to adjust the BC clinical approach, the briefly treated evidences here represent a small drop in the ocean. However, they provide, at the same time, a stable basis from which to develop new and more specific approaches and treatments for the management of BC, improving clinical outcomes and reducing toxicity with the aim to make BC therapies increasingly personalized.
